# The challenges of managing patients with cancer in the workplace: Needs, opportunities and perspectives of occupational physicians

**DOI:** 10.1371/journal.pone.0288739

**Published:** 2023-07-27

**Authors:** Bruna Maria Rondinone, Luca Fontana, Giuliana Buresti, Mauro Fedele, Grazia Fortuna, Sergio Iavicoli, Maria Giuseppina Lecce, Benedetta Persechino

**Affiliations:** 1 Department of Occupational and Environmental Medicine, Epidemiology and Hygiene, Italian Workers’ Compensation Authority (INAIL), Rome, Italy; 2 Department of Public Health, Section of Occupational Medicine, University of Naples Federico II, Naples, Italy; 3 Ministry of Health, Rome, Italy; Local Health Authority Caserta: Azienda Sanitaria Locale Caserta, ITALY

## Abstract

Cancer is a global major public health problem since it is a leading cause of death, accounting for nearly 10 million deaths in 2020 worldwide and the most recent epidemiological data suggested that its global impact is growing significantly. In this context, cancer survivors have to live for a long time often in a condition of disability due to the long-term consequences, both physical and psychological. These difficulties can seriously impair their working ability, limiting the employability. In this context, the occupational physician plays a key role in the implementation and enforcement of measures to support the workers affected by cancer, to address issues such as the information on health promotion, the analysis of work capacity and the management of disability at work and also promoting a timely and effective return to work and preserving their employability. Therefore, the aim of this study was to gather useful information to support the occupational physicians in the management of workers affected by cancer, through a survey on 157 Italian occupational physicians. Based on the interviewees’ opinions, the most useful occupational safety and health professionals in terms of job retention and preservation of workers affected by cancer are the employers and the occupational physicians themselves, whose role is crucial in identifying and applying the most effective reasonable accommodations that should be provided to the workers affected by cancer. The provision of these accommodations take place on the occasion of mandatory health surveillance medical examination to which the worker affected by cancer is subjected when he returns to work. Results on training and information needs showed that the management of the workers affected by cancer is essentially centered on an appropriate fitness for work judgment and on the correct performance of health surveillance. However, an effective and successful management model should be based on a multidisciplinary and integrated approach that, from the earliest stages of the disease, involves the occupational physicians and employers.

## Introduction

Cancer is a global major public health problem since it is a leading cause of death, accounting for nearly 10 million deaths in 2020 worldwide [1]. Recently, the data provided by the World Health Organization, based on the GLOBOCAN estimates, indicated that the annual incidence of new cancer cases is around 19 million [2], thus showing a significant upward trend so much so that for several years now there has been open talk of a cancer pandemic [3, 4]. Considering these figures, it is clear that cancer has an impressive economic, social and health impact on both individuals and the communities in which cancer patients live [5, 6]. In this regard, it should be considered that, apart from the obvious health issues related to high mortality rates and often extremely demanding and sometimes disabling therapeutic treatments, in many cases cancer patients (and their families) experience high rates of financial hardship, if not outright catastrophes, as cancer treatment becomes increasingly expensive but also as a result of the fact that the disease seriously impairs the working capacity of these subjects who, being largely limited in their employability, might suddenly no longer be able to support themselves and their families financially [7–9].

Indeed, nearly half of people diagnosed with cancer are in the middle of their traditional working age (between 20 and 65 years) [10] and physical, emotional, cognitive fatigue, along with numerous other cancer-related symptoms and impairments (e.g. emotional strain, depression, anxiety, pain, reduced attention and memory) severely interfere with people’s ability to work to such an extent that has been demonstrated how a cancer diagnosis (with all that goes with it) compromises patients’ employment status, constraints their job opportunities, work participation and work ability [11–17]. In this context, the main determinants of these occupational difficulties are to be found in the high rate of absenteeism in the phase immediately following the diagnosis and in the working difficulties due to psycho-physical disabilities that affect the return to work (RTW) and often lead to higher unemployment rates [18–22]. In greater detail, the overall risk of unemployment is 1.4 times higher in cancer patients than in non-cancer patients, while their RTW rate is about 63.5% [23, 24]. Therefore, it is quite clear that policies, strategies and practical interventions aimed at supporting an earlier RTW and employability of cancer patients and survivors are a priority within the complex issue of cancer control [7, 14].

In this regard, various types of strategies and practical interventions have been described in the literature that aim to support the workers affected by cancer (WAC) with regard to the above-mentioned problems [14]. Among these, the most common are guaranteeing workers specific and reasonable accommodations, reducing their working hours (or otherwise ensuring more flexible working hours), providing paid sick leave, modifying workload, changing duties or working activities, informing about psycho-educational interventions and rehabilitation services [14]. Interestingly, most, if not all, of the proposed examples have in common that their implementation involves the occupational physicians (OPs), who therefore plays a key role in the design, implementation and enforcement of these measures. On the other hand, a modern OP should be in effect a global consultant for both employers and employees and then he should be able to address issues such as the information on health promotion, the analysis of work capacity and the management of disability at work [25]. In any case, even if we want to neglect these OPs’ skills and expertise but take into consideration only his main professional activity, that is the evaluation of fitness for work, it should still be kept in mind that in this process the OPs should not only take into account potential workplace-related threats to workers’ health but they should also consider any diseases, health issues or disabilities that could prevent the worker from doing his job safely and effectively [26, 27].

Therefore, nowadays an emerging body of evidence supports what the occupational safety and health professionals, OPs, as well as WAC, have long recognized: that OPs’ role, competencies and skills are essential to identify and making effective interventions to support and sustain WAC in the workplaces [14]. However, nevertheless, there is still a lack of information on OPs professional activity, information demands and updating needs related to the management of WAC. Thus, this study, conducting a survey on Italian OPs, aimed to deepen the current knowledge of these topics in order to obtain useful information to define and implement instruments, practices, policies and interventions that should be able to support the OPs in the management of these patients within the workplaces.

## Materials and methods

### Participants and sample selection

A 4 yr postgraduate training course in occupational medicine, in accordance with the Decree Law no. 81/2008, is requested to practice the profession of OP in Italy. Alternatively, also specialists in forensic medicine or hygiene and preventive medicine who attended a 2nd-level university master course or lecturers (with a proven period of teaching) in occupational medicine, in industrial toxicology or hygiene and similar teaching courses, can perform the OP profession. Moreover, this role can also be carried out by physicians in possession of the authorization pursuant to article 55 of Decree Law no. 277/1991 (these are physicians who, at the time of entry into force of this law, had already carried out the OP profession). OPs in Italy are mainly freelance practitioners who work with employers and/or companies or with private occupational health centers; they could be, also, employed in the Local Public Health Authority (Department for Prevention and Occupational Health and Safety) of the National Health System or work within public institutions and universities. All Italian OPs must be enrolled in the national register of OPs of the Italian Ministry of Health [27].

This study enrolled a sample of 157 Italian OPs. A convenience sampling approach was used, contacting the OPs who had participated on previous surveys conducted by the Italian Workers’ Compensation Authority (INAIL). The possession of the legal requirements to perform the professional activity of OP in Italy and the enrollment in the national register of OPs of the Italian Ministry of Health at the time when the study was conducted, were the inclusion criteria to be included in the survey. An electronic form or a mailed version of the questionnaire, a form for informed consent, a cover letter, which explained the purposes of the study were sent to the OPs. All non-respondents were sent a reminder letter approximately one month after the first invitation in order to encourage them to complete and return the questionnaire. The filled-in questionnaires returned to INAIL were coded and the data were entered into an electronic file.

The study is in accordance with the ethical standards of the institutional and/or national research committee and with the guidelines of the 1964 Declaration of Helsinki and its amendments. Due to the anonymous of data collected, observational design of the study and the absence of patient clinical data, ethical review and approval were waived for this study, in accordance with the Italian law. Indeed, in Italy, the combined provisions of Decree Law 158/2012 and its conversion Law 189/2012 and the Ministry of Health Decree of 8 February 2013, define ethics committees as independent bodies to which are attributed competencies relating to clinical trials of medicines, issues on the use of medicines and medical devices, issues on the use of surgical and clinical procedures or relating to the study of food products on humans generally referred, by international practice, to the assessments of the committees, in order to ensure the protection of the rights, safety and well-being of persons undergoing trials and to provide public assurance of such protection. This study investigated, through the administration of a questionnaire, the point of view of OPs referring to professional activity, information demands and their updating needs related to any critical issue in the management of WAC. Therefore, since it does not concern experimentation of any kind, it does not fall under the above-mentioned cases for which approval of an ethics committee is required.

### Questionnaire

According to the aims of the present study, a structured questionnaire was developed in order to obtain valuable information regarding different issues. The items and questions could not directly be derived from any already existing questionnaire but were designed on the base of the results of previous experience [27, 28]. The questionnaire consisted of 36 close-ended questions organized in 4 sections: 1) the OPs demographic and professional characteristics (10 items) (e.g. gender, age, legal requirements to perform the activity of OP, etc.); 2) OPs professional activity, especially referring to the health surveillance system and the evaluation of fitness for work in both WAC and long-term cancer survivors (LTCS) (7 items for the management of WAC and 7 items for LTCS); 3) the level of knowledge of the legislation regarding the social security benefits, the welfare benefits, the “targeted placement” (Law no. 68/1999 and Prime Ministerial Decree no. 91/2000) and the procedures to ask “rights due” (9 items); 4) the OPs information demands and updating needs on issues related to cancer and work (3 items). Different scales were used in the 3^rd^ and 4^th^ sections of the questionnaire: 0–5 scales (0 = no knowledge; 5 = full knowledge) investigated the level of knowledge of issues regarding social security benefits, welfare benefits, etc.; 0–10 scales (0 = useless; 10 = completely useful) explored the level of utility of some aspects (involvement of different types of figures in the management of WAC, various types of medical visits for the maintenance of the job of the WAC, etc.). A five-point Likert scale (1 = completely disagree; 5 = completely agree) was also used to measure the level of agreement with some items, based on the experience as OPs.

In this context, in order to facilitate the understanding of the information here reported and the correct interpretation of the results, we find it useful to make a few clarifications on the main concepts of health surveillance and on the legislative framework governing the management of cancer patients (Sections 2 and 3 of the S1 Appendix). Regarding the provision of health surveillance medical examinations, the normative framework of reference is the aforementioned Decree Law no. 81/2008, which, in the context of health surveillance of workers exposed to specific occupational risk factors, provides for the implementation of different types of medical examinations. Briefly, in accordance with the law, workers undergo these health checks as soon as they are hired by the employer and then before they are exposed to occupational hazards (preventive medical examination) and then usually once a year (periodic medical examination). In addition, workers must also be required to undergo a medical examination when they change job role (medical examination for job role change) or when they return to work after a period of uninterrupted absence (due to illness) of more than 60 days (RTW medical examination). Beyond these instances, the worker may still request, at any time, that his or her employer provide him or her with a health surveillance medical examination (visit requested by worker). From this point on, for the sake of conciseness and simplicity, we will comprehensively include preventive, periodic, job role change and RTW medical examinations under the term "mandatory health surveillance" to differentiate them from the health surveillance medical examination requested by the worker.

As far as the legislative aspects are concerned, it should be underlined that in Italy the management of cancer patients, their social protection and welfare issues are regulated by various laws. For example, in matter of social protection, the Law no. 222 of 1984 establishes the criteria for the granting of “ordinary disability allowance” (an economic emolument which is provided, every three years in three years, to the worker whose working capacity is permanently reduced by less than a third due to physical or mental illness), “disability pension” (a permanent economic annuity which is recognized to workers who, due to disease/physical or mental impairment, are absolutely and permanently unable to carry out any work activity) and the “monthly allowance for ongoing personal assistance” (an economic benefit that integrates the previous ones and which is granted to those workers who need continuous assistance as they are unable to carry out normal and daily personal activities. On the other hand, similar welfare benefits such as “attendance allowance” and “disability pension or allowance” are disciplined by Law no.118 of 1971 and 18 of 1980. Finally, due to its relevance to the issues covered in this article, particular mention should be made of targeted placement, which is a tool by which disabled workers (i.e., civilian disabled with a reduction in work capacity equal or exceeding 46%) are guaranteed a sort of preferential lane to be hired as protected categories.

A preliminary pilot test was conducted on a small sample (n = 20) of OPs to collect feedback on the length, content, clarity and comprehensibility, face validity and acceptance by the interviewees. Suggestions and observations gathered were considered to develop the final version of the questionnaire. The responses included no personal identifiers such as name or date of birth and all information was kept confidential (S1 Appendix).

### Statistical data analysis

Statistical analysis was performed using SPSS software version 22. For categorical and Likert scale variables, percentages and frequencies were calculated on the total sample and, at a greater level of detail, contingency tables were employed to display the frequency distribution of the variables in the subsets generated by demographic variables, in order to highlight any peculiarities. For 0–5 and 0–10 scales, mean scores and standard deviations were calculated.

## Results

### Sample description

Demographic and professional information of Italian OPs enrolled in the survey are reported in Table 1. Most of the respondents were male (65.0%), aged between 55 and 64 years (35.3%), coming from South and Islands (33.3%). As regards the legal requirements to carry out professional activity as an OP in Italy, 85.4% of the respondents were specialized in occupational medicine, 3.8% in hygiene and preventive medicine, whereas 10.8% were in possession of the authorization pursuant to article 55 of Law Decree no. 277/1991. Most surveyed OPs were self-employed (81.2%), carried out their activity of OP in private companies (61.0%) and on a total number of workers between 1001 and 1500 (20.5%).

**Table 1:** Sample description.

Gender	N.	%
Male	102	65.0
Female	55	35.0
**Age**	**N.**	**%**
< 35 yr	4	2.6
35–44 yr	38	24.4
45–54 yr	47	30.1
55–64 yr	55	35.3
≥ 65 yr	12	7.7
**Geographical area of residence**	**N.**	**%**
Northwest Italy	41	26.3
Northeast Italy	28	17.9
Middle Italy	35	22.4
South and Islands	52	33.3
**Legal requirements to perform OP profession**	**N.**	**%**
Specialty in OM	134	85.4
Authorization pursuant to article 55 of Decree Law no. 277/1991	17	10.8
Specialty in hygiene and preventive medicine	6	3.8
**OP profession as…**	**N.**	**%**
Self-employed	125	81.2
Employee	29	18.8
**Activity of OP carried out in…**	**N.**	**%** ^**a**^
University	13	6.3
Local Health Authority (ASL)	18	8.8
Public company	48	23.4
Private company	125	61.0
Other	1	0.5
**Total number of workers examined as OP in a year**	**N.**	**%**
≤ 200	13	8.9
201–500	24	16.4
501–800	29	19.9
801–1000	29	19.1
1001–1500	30	20.5
> 1500	21	14.4

^a^Multiple choice. Percentages of answers.

### OPs professional activity in managing workers affected by cancer and long-term cancer survivors

In Table 2 we reported the main findings related to the management of WAC and LTCS by Italian OPs. Most of the participants had to deal with at least one of these workers in the 5 years prior to the survey (82.9% managed WACs; 75.8% managed LTCS). Among these, the management took place in occasion of mandatory health surveillance (respectively: 66.5% and 82.3%). Among workers dealt with mandatory health surveillance, most of them were exposed to several risk factors, mainly visual display units (respectively: 22.0% and 34.8%). 27.8% of respondents (in case of the management of WACs) and 15.1% of OPs who managed LTCS declared that they need to carry out further diagnostic insights at the time of the visit, in order to get more information on workers previously diagnosed with cancer. In most cases, the determination of the fitness for work judgment of a WAC is not a critical issue for the surveyed OPs, but it should be noted, however, that in a good percentage (43.7%), OPs must establish specific prescriptions and/or limitations (related to the performance of work activities and tasks) to ensure that WAC have reasonable accommodations that are in turn capable of supporting their working ability; 24.4% towards LTCS. These difficulties are mainly due to the typology of risk factors (49.1% for WAC and 51.1% for LTCS).

**Table 2:** Management of workers affected by cancer and long-term cancer survivors, in the last 5 years.

	OPs who have/not managed WAC	OPs who have/not managed LTCS
	N (%)	N (%)
No	26 (16.6)	38 (24.2)
Yes	126 (82.9)	119 (75.8)
	**OPs who have managed WAC** (N = 126)	**OPs who have/not managed LTCS** (N = 119)
**Which organ/system is affected?** ^**a**^	**N (%)**	**N (%)**
Breast	94 (43.1)	98 (63.6)
Uterus	31 (14.2)	18 (11.7)
Colon	25 (11.5)	14 (9.1)
Lung	14 (6.4)	0 (0.0)
Blood and lymphatic	13 (6.0)	15 (9.7)
Stomach	11 (5.0)	1 (0.6)
Prostate	11 (5.0)	3 (1.9)
Skin	10 (4.6)	2 (1.3)
Bladder	5 (2.3)	3 (1.9)
Kidneys	4 (1.8)	0 (0.0)
**The management took place in occasion of…** ^**a**^	**N (%)**	**N (%)**
Mandatory health surveillance	115 (66.5)	102 (82.3)
Visit requested by the worker	49 (28.3)	19 (15.3)
Counselling	9 (5.2)	3 (2.4)
**If took place during the mandatory health surveillance, to which risk was the worker exposed to?** ^**a**^	**N (%)**	**N (%)**
Visual Display Units	57 (22.0)	56 (34.8)
Chemical agents	53 (20.5)	29 (18.0)
Manual handling of loads	41 (15.8)	31 (19.3)
Noise	29 (11.2)	11 (6.8)
Biological agents	28 (10.8)	18 (11.2)
Upper Limb Repetitive Movements	14 (5.4)	10 (6.2)
Night job	14 (5.4)	3 (1.9)
Carcinogens	11 (4.2)	1 (0.6)
Vibrations	9 (3.5)	2 (1.2)
Other	3 (1.2)	0 (0.0)
**Did you need diagnostic investigations when you visited workers previously diagnosed with cancer?**	**N (%)**	**N (%)**
No	91 (72.2)	101 (84.9)
Yes	35 (27.8)	18 (15.1)
**Have you had any difficulty in issuing a fitness for work judgment without limitations or prescriptions when you visited workers previously diagnosed with cancer?**	**N (%)**	**N (%)**
No	71 (56.3)	90 (75.6)
Yes	55 (43.7)	29 (24.4)
**The difficulties were mainly due to:** ^**a**^	**N (%)**	**N (%)**
Typology of risk factors	53 (49.1)	24 (51.1)
Ergonomic nature of workstation	23 (21.3)	8 (17.0)
Work environment	19 (17.6)	10 (21.3)
Working hours	13 (12.0)	5 (10.6)

^a^Multiple choice question. Percentages of answers.

### Level of knowledge of legislation from the point of view of OPs

In order to deepen the current level of knowledge of OPs related to legislation, we calculated mean scores and standard deviation on a 0–5 scale (0 = no knowledge; 5 = full knowledge) (Table 3).

**Table 3:** OP’s level of knowledge regarding different issues of legislation rated on a scale variable from 0 = no knowledge to 5 = full knowledge.

	Mean score (SD)
**Social security benefits**	
Disability pension	1.79 (1.02)
Monthly allowance for ongoing personal assistance	1.77 (1.03)
Ordinary disability allowance	1.74 (1.00)
**Welfare benefits**	
Attendance allowance	2.91 (1.22)
Disability pension	2.66 (1.15)
Disability allowance	2.64 (1.15)
**Targeted placement** (Law no. 68/1999 **and** Prime Ministerial Decree no. 91/2000)	
Beneficiaries	1.22 (0.89)
Methods of assessment	1.07 (0.85)
Functional diagnosis	1.07 (0.84)
Workers who became disable after hiring	0.99 (0.87)
**Procedures for requesting the following rights due by workers affected by cancer**	
Absence for life-saving therapy	3.25 (1.09)
Paid work permits	3.07 (1.12)
Exemption from night work	2.04 (0.91)
Part-time	1.99 (0.93)
Unpaid leave	1.49 (0.82)
Early retirement	1.24 (0.69)
Telework	1.07 (0.60)
Choice of place of work and transfer	0.85 (0.67)

In the field of social security benefits, level of knowledge is lower than 2.00 for all issues (ordinary disability allowance, disability pensions and monthly allowance for ongoing personal assistance). Also, for targeted placement issues, mean scores are around 1.00. For welfare benefits, level of knowledge is higher than 2.50 but lower than 3.00 for all issues (disability allowance, disability pension and attendance allowance). For topics regarding the procedures for requesting the rights due by WAC, best known topic is the absence for life-saving therapy (mean score = 3.25), whereas less known topic is the choice of place of work and transfer (mean score = 0.85).

In Table 4, levels of agree/disagree about a list of items related legislation are reported, through percentages of responses.

**Table 4:** Level of agree/disagree with some items, according to the experience as occupational physician.

	Completely and mostly disagree N (%)	Slightly agree N (%)	Mostly and completely agree N (%)
The recognition of the status of "serious handicap" determines, anyway, the formulation of an unfitness for work judgment	130 (82.8%)	26 (16.6%)	1 (0.6%)
The recognition of the status of "100% civil invalidity" in any case precludes the performance of any work activity	112 (71.8%)	40 (25.6%)	4 (2.5)
The adoption of adequate "reasonable accommodations" at work favors the maintenance of worker’s with neoplastic disease workplace	5 (3.1%)	72 (45.9%)	80 (51.0%)
The role of the OP is important in the choice of "reasonable accommodations" at work	3 (1.9%)	43 (27.4%)	111 (70.7%)
In case of job placement through "targeted placement" of a patient with oncological pathology, the OP must be informed	10 (6.4%)	63 (40.1%)	84 (53.5%)
The Risk Assessment Document must provide for specific procedures in case of inclusion in the workplace of a person with disability	8 (5.1%)	80 (51.0%)	69 (43.9%)
The contact with level II structures of the National Health System (NHS) is important for managing the fitness of a worker with oncological pathology	4 (2.5%)	35 (22.3%)	118 (75.2)

Through a 0–10 scale (0 = useless; 10 = completely useful) we measured the level of utility, by the OP’s point of view, of aspects regarding the involvement of different figures in the management of WAC and different types of medical visits for the maintenance of the job.

Regarding the various types of figures involved in the WAC’s management, the employer and the OPs themselves are considered the most useful Occupational Health and Safety (OSH) professionals (mean values: 7.33 and 6.28, respectively). Other figures, such as the Health and Safety Manager, the Health and Safety Representative and the worker reach a lower score, respectively, 4.26, 2.29 and 2.29. For the same purpose, the most useful visits are the one prior to RTW after 60 continuous days of absence for health reasons (6.93), the visit requested by the worker (6.87), the visit for change of job (6.69), and the periodic visit (6.11).

### OPs information demands and updating needs

**Fig 1:**
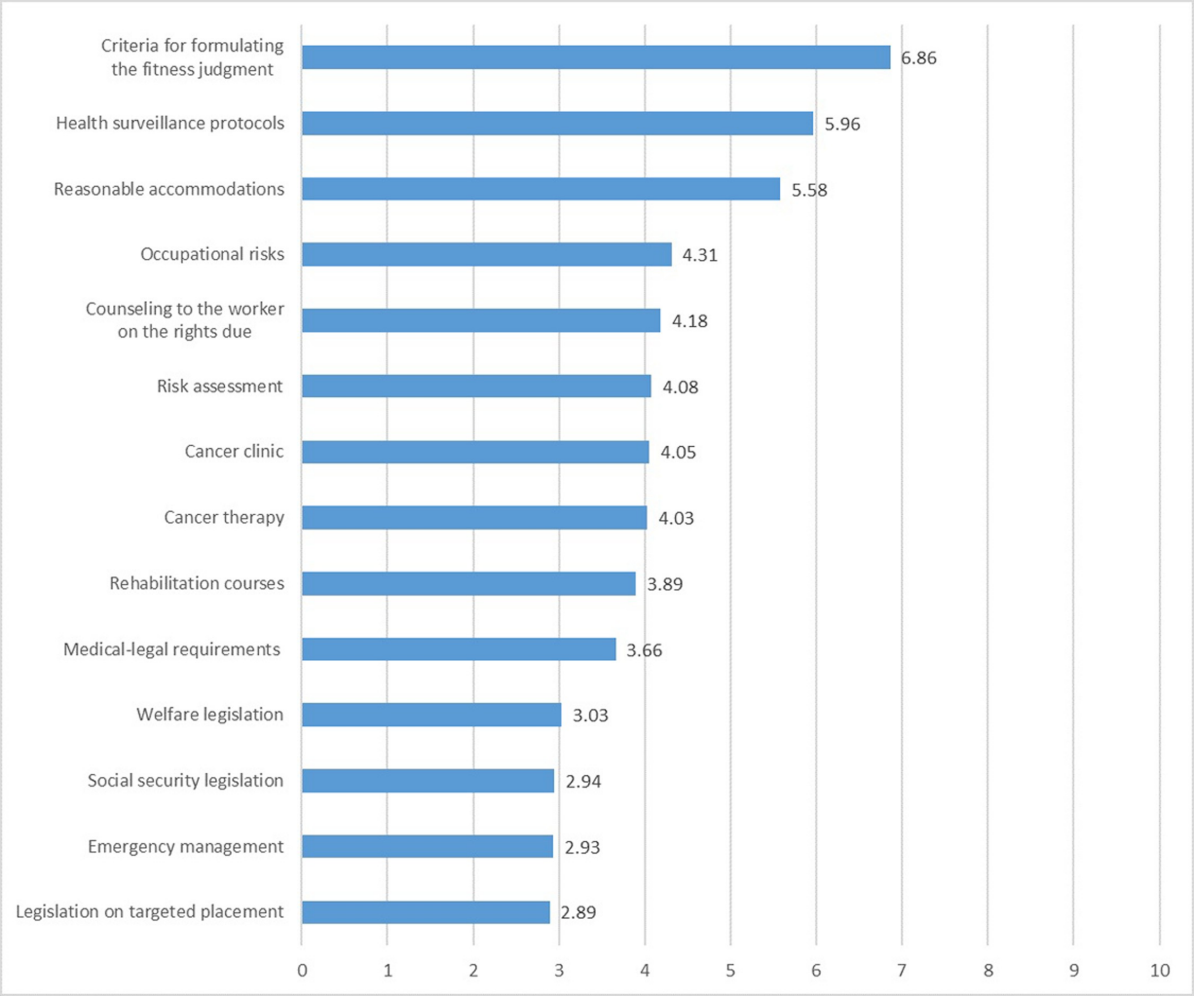
Usefulness of training needs relating to cancer and work (0 = useless to 10 = completely useful).

This paragraph shows results on information demands and updating training needs related to the management of WAC, by the OP’s point of view. 42.0% of the sample agreed (mostly and completely) with the statement that specific training in the field of cancer and work is important for the OP. On a 0–10 scale (0 = useless; 10 = completely useful), a list of topics that would be necessary for the training needs in terms of neoplastic diseases and work is shown in decreasing order, from the most useful to the least useful. The most useful are criteria for formulating the fitness judgment (6.86), the health surveillance protocols (5.96) and the reasonable accommodations (5.58) (Fig 1).

Moreover, training and update needs is also compared between OPs that managed WAC or long-term cancer survivors and OPs who did not, in the 5 years prior to the survey. Scores for training needs for OPs that did not manage both WAC and long-term cancer survivors, is always higher than the OPs that managed these types of workers, except for the issue “counseling to the worker on the rights due” (S1 Fig).

### Focus on OPs dealing with workers affected by breast cancer

A deeper analysis was carried out on the sub-samples of OPs who managed workers affected by breast cancer (n = 94 OPs) and long-term breast cancer survivors (n = 98 OPs). S2 Fig shows the results related to the risks to which workers are exposed, separately for workers affected by breast cancer and long-term breast cancer survivors. Regarding the fitness for work judgment without limitations or prescriptions, 45.7% of OPs dealing with WAC had some difficulties in issuing it. While this percentage decreased to 22.4% in case of OPs dealing with long-term breast cancer survivors. In both cases, these difficulties are mainly due to the typology of risk factors, respectively for 49.4% and 51.5%.

## Discussion

The present survey has provided rather interesting findings concerning several features of the professional activity of Italian OPs who are often dealing with the delicate issue of expressing the fitness for work judgment with respect to workers suffering from cancer diseases. This extremely challenging topic requires adequate knowledge of the legislative framework of managing these patients and continuous updating of the skills and abilities of OPs who, as confirmed by the results of our research, should play a key role in improving work ability, sustaining work capacity, and fostering the employability of WAC. Indeed, our data confirm this as interviewed OPs consider that the most useful occupational safety and health professionals in terms of job retention and preservation of WAC are the employers and the OPs themselves. On the other hand, our results on the OPs professional activity suggest that this strong conviction of the study participants is a consequence of the fact that OPs are well aware that their role is crucial in identifying and applying in the workplace the most appropriate and effective special and reasonable accommodations that the occupational health and safety system must be able to provide to the WAC (Table 4). This topic is essential in the management of these individuals as evidence showed that several symptoms of the disease (but also of short- and long-term therapeutic treatments) such as fatigue, emotional strain, anxiety, depression, pain, decreased cognitive functions (especially regarding attention and memory) cause a significant decrease in functioning at work which, in the long run, makes it difficult, if not impossible, for them to continue to perform their usual tasks adequately and safely [10–17, 29, 30].

In this regard, it is worth remembering that the OPs, being aware of the psycho-physical conditions of the cancer patient and, at the same time, knowing what occupational risk factors the WAC is exposed to (e.g., visual display units, chemical agents, manual handling of heavy loads, noise, biological agents, upper limb repetitive movements; see Tables 2 and 3), can weigh and balance these different needs (personal health status *vs* physical, cognitive, and interpersonal work demands), matching them and thus finding the right balance that allows these people to continue working in a satisfactory and lasting way [27]. In this regard, it is interesting to note that the percentage of WAC exposed to “Visual Display Units” is 10 points lower than the same item for LTCS, thus suggesting that probably a common management strategy for these workers among surveyed OPs is to assign them to office administrative activities. It is therefore quite clear that the OPs is in a privileged position to establish what are the reasonable accommodations that the WAC should have, and usually these indications take the form of prescriptions or limitations to the fitness for work regarding specific tasks [27, 28]. In most cases, the provision of these accommodations taken place on the occasion of mandatory health surveillance medical examination to which the WAC is subjected when he returns to work (RTW health surveillance) which, not surprisingly, is indicated by the surveyed OPs as the most important and useful one in the management of these workers (Table 2). From the OP’s perspective this result is absolutely understandable since, as long as the cancer patient does not return to work and is entrusted to the care of other health specialists such as the oncologist or physiotherapist, they have no obligation towards him. However, it has long been known that one of the main barriers that greatly hinder an effective and early RTW of cancer patients (and therefore have a strong impact also on financial and employment outcomes) is represented by the lack of involvement in this complex process of the OPs and employers [31–34]. Furthermore, even where such involvement is carried out, it is largely inadequate, poor and defective, especially in terms of communication between the OPs and both other health professionals and patients [34] or in the analysis of the workload and job demands [31]. Nevertheless, even the most recent literature data continue to underline these shortcomings and the consequent need to build modern and integrated vocational and occupational rehabilitation paths that should be multidisciplinary contemplating an early intervention by the OPs, even in the early stages of diagnosis and therapy [14, 33].

On the contrary, the current model of management of the cancer patient would seem to be split into two segments divided by a thin red line represented by the RTW, before which the cancer patient is essentially managed by the oncologist (and/or other health professionals) and only after his/her RTW becomes primarily a responsibility of the OPs [34]. This state of affairs is actually also suggested by the results of our survey on information demands and updating needs (Fig 1), which showed that the management of the oncological worker by OPs is essentially centered on the expression of an appropriate fitness for work judgment and on the correct performance of health surveillance. Indeed, survey participants emphasized their need to be updated on issues such as the criteria for formulating the fitness for work, the definition of adequate health surveillance protocols and the identification of the best reasonable accommodations to provide to WAC, whereas other topics such as updating on rehabilitation courses or on medico-legal requirements were assessed by the occupational physicians as being of little use (Fig 1) [14]. In this regard, these findings seemed rather surprising, especially considering the low level of knowledge declared by the respondents about different legislation issues such as social security or welfare benefits and the targeted placement (Table 3). Actually, the data on the level of knowledge of legislative aspects also seemed rather surprising, especially in view of the fact that almost half of the sample was over 55 years old. From these individuals, who are specialists in the field and are supposed to have a long career and experience behind them, one would expect completely different results. Therefore, although these data are a little discouraging, at the same time, by identifying a critical issue, they can be helpful in stimulating the design and implementation of targeted training and updating courses on this matter. Indeed, also in this case, it is likely that the answers provided by OPs are driven by a health surveillance-centric vision of the WAC management and therefore it is plausible to assume they deemed that these issues are not of interest to them but rather are substantially a competence of the employer and human resources manager or the worker himself. Although, this reasoning basically reflects the reality of things and is all in all correct, in our opinion, the most interesting aspect suggested by this data is, once again, related to the fact that the practical management of WAC in the workplace is flawed and fragmented. In other words, both the RTW pathway and the management of the oncological patient in the workplace are severely hampered by the lack of (or at least poor) communication and interaction between the different healthcare and non-healthcare figures who, in various capacities and at different times, are involved in the care of the WAC. On the whole, it is as if the management of these subjects were based on a watertight compartmentalized model in which each actor involved in the process focuses on his/her own specific area of interest without bothering to interact with the stakeholders who are upstream and downstream of the rehabilitation and reintegration into work pathway.

However, as already mentioned above, all available literature data showed that an effective and successful management model, which is actually able to support an early RTW of the cancer patients and then implement their ability to work in the medium to long term, should be based on a multidisciplinary and integrated approach that, from the earliest stages of the disease, involves the OPs and employers [14, 35–37]. In this context, while keeping in mind the specificities and peculiarities of the subject matter, in order to make OPs (but more generally all stakeholders involved) aware of the need to build and actively participate in all phases of this management model, it might be useful to refer to the fundamental principles of the Total Worker Health^®^ (TWH) approach developed by the National Institute for Occupational Safety and Health [38]. Indeed, this model aims to identify, implement and disseminate policies and intervention strategies that comprehensively protect the safety and health of workers by considering and evaluating both occupational and/or work-related risk factors and personal health conditions and/or lifestyle factors and the possible interactions and reciprocal influences between all these elements [39]. In this regard, the basic principles of TWH, recalling the need for a close collaboration between OP, employer, human resources manager and other health specialists, and at the same time taking into account the interactions between occupational risk factors (or work-related ones) and the worker’s personal health conditions, would also seem to fully meet the requirements of an adequate and effective management of the cancer patient in the workplace.

Although this survey has provided interesting results, it is necessary to underline some limitations: first of all the lack of data regarding the opinion and the viewpoint of WAC is the main limitation of the study. Another possible limitation is related to the chosen tool (self-administered questionnaire) to carry out the survey which could be associated with a lower involvement of participants or difficulties in understanding and filling in the questionnaire. However, it should be noted that we tried to solve these issues providing to OPs a cover letter that described the research aims and supplied appropriate instructions on the proper understanding and filling of the tool. Furthermore, a selection bias is possible since the study population was made up of OPs who had already collaborated or participated on previous surveys carried out by INAIL.

## Conclusions

The results obtained by this survey indicated that OPs are frequently involved in the health surveillance of WAC and that this interaction is often characterized by difficulties in issuing the fitness for work due to the reduced working capacity of these workers who prevents them from carrying out some specific working tasks (especially when these involve exposure to certain occupational risk factors). Considering the most recent epidemiological data and the progressive aging of the workforce, it is likely that in the near future the number of workers suffering from cancer with whom the OPs will have to deal with will increase significantly. Therefore, it is necessary to adequately inform the OPs and provide them with innovative operational strategies and current practical tools that allow them to actively collaborate with the other OSH system figures in identifying the reasonable accommodations to be provided to the WAC in order to increase their resilience in the workplaces.

In this regard, in order to obtain the best possible results, our data agree with the evidence already available in indicating the opportunity to increase information flows and collaboration between all the stakeholders involved. In detail, with regard to OPs, the achievement of this objective entails a paradigm shift, that is the transition from a management model primarily focused on health surveillance to a broader, integrated and multidisciplinary approach whose purpose is to ensure (from the moment of diagnosis to RTW and beyond) the complete well-being of the workers and not only their fitness for work.

## Supporting information

S1 Appendix Questionnaire. (DOC)

S1 Data (XLSX)

S1 Fig Level of usefulness of training and update needs of some issues, expressed by Occupational Physicians (OPs) managing/not managing workers affected by cancer (WAC) and long-term cancer survivors (LTCS) (0 = useless to 10 = completely useful). (TIF)

S2 Fig Risks to which workers are exposed according to Occupational Physicians (OPs) managing workers affected by breast cancer (n = 94) and OPs managing long-term breast cancer survivors (n = 98). Multiple choice question. Percentages of answers.
(TIF)
