# Supplementary material for: The challenges of managing patients with cancer in the workplace: Needs, opportunities and perspectives of occupational physicians
Source: PLoS One. 2023 Jul 27;18(7):e0288739. doi: 10.1371/journal.pone.0288739 (PMC10374089; doi:10.1371/journal.pone.0288739)
Supplement: S1 Appendix — (DOC) [file pone.0288739.s001.doc]

**S1 Appendix. Questionnaire.**

# Section A. Occupational Physicians individual demographics and professional characteristic

# Gender

# Male

# Female

# Year of birth ___________

# Region of residence ______________

# Medicine graduation year ______________

# What is the legal requirements that you have to perform Occupational Physician profession?

# Specialty in occupational medicine

# Specialty in forensics medicine

# Specialty in hygiene and preventive medicine

# Authorization pursuant to article 55 of Legislative Decree n. 277/91

# Specialty year _______________

# Starting year of the activity as Occupational Physician _____________

# Do you practice the Occupational Physician profession as:

# Self-employed

# Employee

# Occupational Physician activity is carried out at:

# University

# Local Health Authority (ASL)

# Public company

# Private company

# Other

# Doing the Occupational Physician profession, how many workers do you globally visit?

# ≤200

# 201-500

# 501-800

# 801-1000

# 1001-1500

# >1500

# Section B. Health surveillance system and evaluation of fitness for work in workers affected by cancer and long-term cancer survivors

# Have you ever managed, in the last 5 years, workers affected by cancer?

# Yes

# No

# If yes, which organ/system is affected? (Multiple choice question)

# Breast

# Lung

# Colon

# Stomach

# Kidneys

# Haemolymph poietic

# Uterus

# Central Nervous System (CNS)

# Prostate

# Bladder

# Skin

# Other

# If yes, the management took place in occasion of…

# Mandatory health surveillance

# Visit requested by the worker

# Counseling

# If the management took place during the mandatory health surveillance, to which risk was the worker exposed to? (Multiple choice question)

# Chemical agents

# Carcinogens

# Biological agents

# Noise

# Vibrations

# Manual handling of loads

# Upper limb repetitive movements

# Visual display units

# Night job

# Other

# Did you need further diagnostic investigations at the time of the visit of workers previously diagnosed with cancer?

# Yes

# No

# Have you had any difficulty in issuing a fitness for work judgment without limitations or prescriptions?

# Yes

# No

# If yes, difficulties were mainly due to: (Multiple choice question)

# Typology of risk factors

# Ergonomic nature of workstation

# Work environment

# Equipment and working machinery

# Working hours

# Other

# Have you ever managed, in the last 5 years, long-term cancer survivors?

# Yes

# No

# If yes, which organ/system is affected? (Multiple choice question)

# Breast

# Lung

# Colon

# Stomach

# Kidneys

# Haemolymph poietic

# Uterus

# Central Nervous System (CNS)

# Prostate

# Bladder

# Skin

# Other

# If yes, the management took place in occasion of…

# Mandatory health surveillance

# Visit requested by the worker

# Counseling

# If the management took place during the mandatory health surveillance, to which risk was the worker exposed to? (Multiple choice question)

# Chemical agents

# Carcinogens

# Biological agents

# Noise

# Vibrations

# Manual handling of loads

# Upper limb repetitive movements

# Visual display units

# Night job

# Other

# Did you need further diagnostic investigations at the time of the visit?

# Yes

# No

# Have you had any difficulty in issuing a fitness for work judgment without limitations or prescriptions?

# Yes

# No

# If yes, difficulties were mainly due to: (Multiple choice question)

# Typology of risk factors

# Ergonomic nature of workstation

# Work environment

# Equipment and working machinery

# Working hours

# Other

**SECTION C. Level of knowledge of the legislation regarding the management of workers affected by cancer**

# Which is your level of knowledge regarding issues of social security benefits for workers insured by National Social Security Institute? (scale from 0=no knowledge to 5=full knowledge).

|  | **0**  **No knowledge** | **1** | **2** | **3** | **4** | **5**  **Full Knowledge** |
| --- | --- | --- | --- | --- | --- | --- |
| 1. Ordinary disability allowance | 🞏 | 🞏 | 🞏 | 🞏 | 🞏 | 🞏 |
| 1. Disability pension | 🞏 | 🞏 | 🞏 | 🞏 | 🞏 | 🞏 |
| 1. Monthly allowance for ongoing personal assistance | 🞏 | 🞏 | 🞏 | 🞏 | 🞏 | 🞏 |

# Which is your level of knowledge regarding issues of welfare benefits for workers insured by National Social Security Institute? (scale from 0=no knowledge to 5=full knowledge).

|  | **0**  **No knowledge** | **1** | **2** | **3** | **4** | **5**  **Full Knowledge** |
| --- | --- | --- | --- | --- | --- | --- |
| 1. Disability allowance | 🞏 | 🞏 | 🞏 | 🞏 | 🞏 | 🞏 |
| 1. Disability pension | 🞏 | 🞏 | 🞏 | 🞏 | 🞏 | 🞏 |
| 1. Attendance allowance | 🞏 | 🞏 | 🞏 | 🞏 | 🞏 | 🞏 |

# Which is your level of knowledge regarding issues of “targeted placement” (Law no. 68/1999 and Prime Ministerial Decree no. 91/2000)? (scale from 0=no knowledge to 5=full knowledge).

|  | **0**  **No knowledge** | **1** | **2** | **3** | **4** | **5**  **Full Knowledge** |
| --- | --- | --- | --- | --- | --- | --- |
| 1. Beneficiaries | 🞏 | 🞏 | 🞏 | 🞏 | 🞏 | 🞏 |
| 1. Methods of assessment | 🞏 | 🞏 | 🞏 | 🞏 | 🞏 | 🞏 |
| 1. Functional diagnosis | 🞏 | 🞏 | 🞏 | 🞏 | 🞏 | 🞏 |
| 1. Workers who became disable after hiring | 🞏 | 🞏 | 🞏 | 🞏 | 🞏 | 🞏 |

# Are you aware that some National Collective Labor Agreements provide for "rights due" for workers with cancer?

# Yes

# No

# Which is your level of knowledge regarding the procedures for requesting the following “rights due” by workers with neoplastic disease? (scale from 0=no knowledge to 5=full knowledge).

|  | **0**  **No knowledge** | **1** | **2** | **3** | **4** | **5**  **Full Knowledge** |
| --- | --- | --- | --- | --- | --- | --- |
| 1. Paid work permits | 🞏 | 🞏 | 🞏 | 🞏 | 🞏 | 🞏 |
| 1. Unpaid leave | 🞏 | 🞏 | 🞏 | 🞏 | 🞏 | 🞏 |
| 1. Absence for life-saving therapy | 🞏 | 🞏 | 🞏 | 🞏 | 🞏 | 🞏 |
| 1. Early retirement | 🞏 | 🞏 | 🞏 | 🞏 | 🞏 | 🞏 |
| 1. Exemption from night work | 🞏 | 🞏 | 🞏 | 🞏 | 🞏 | 🞏 |
| 1. Part-time | 🞏 | 🞏 | 🞏 | 🞏 | 🞏 | 🞏 |
| 1. Telework | 🞏 | 🞏 | 🞏 | 🞏 | 🞏 | 🞏 |
| 1. Choice of place of work and transfer | 🞏 | 🞏 | 🞏 | 🞏 | 🞏 | 🞏 |

# Which of the listed rules contains the definition of "reasonable accommodation"?

# Law n. 18/09

# Legislative Decree n. 81/08

# Legislative Decree n. 502/92

# Law n. 222/84

# Law n. 18/80

# Based on your experience as Occupational Physician, how much do you agree with the following statements? (scale from 0=completely disagree to 5=completely agree).

|  | **1**  **Completely disagree** | **2** | **3** | **4** | **5**  **Completely agree** |
| --- | --- | --- | --- | --- | --- |
| 1. The recognition of the status of "serious handicap" determines, anyway, the formulation of an unfitness for work judgment | 🞏 | 🞏 | 🞏 | 🞏 | 🞏 |
| 1. The recognition of the status of "100% civil invalidity" in any case precludes the performance of any work activity | 🞏 | 🞏 | 🞏 | 🞏 | 🞏 |
| 1. The adoption of adequate "reasonable accommodations" at work favors the maintenance of worker’s with neoplastic disease workplace | 🞏 | 🞏 | 🞏 | 🞏 | 🞏 |
| 1. The role of the OP is important in the choice of "reasonable accommodations" at work | 🞏 | 🞏 | 🞏 | 🞏 | 🞏 |
| 1. In case of job placement through "targeted placement" of a patient with oncological pathology, the OP must be informed | 🞏 | 🞏 | 🞏 | 🞏 | 🞏 |
| 1. The Risk Assessment Document must provide for specific procedures in case of inclusion in the workplace of a person with disability | 🞏 | 🞏 | 🞏 | 🞏 | 🞏 |
| 1. The contact with level II structures of the National Health System (NHS) is important for managing the fitness of a worker with oncological pathology | 🞏 | 🞏 | 🞏 | 🞏 | 🞏 |

# How useful do you think is the involvement of the following figures in the management of a cancer worker, aimed at maintaining the job? (scale from 0 = useless to 10 = completely useful)

|  | **0**  **Useless** | **1** | **2** | **3** | **4** | **5** | **6** | **7** | **8** | **9** | **10**  **Completely useful** |
| --- | --- | --- | --- | --- | --- | --- | --- | --- | --- | --- | --- |
| 1. Employer | 🞏 | 🞏 | 🞏 | 🞏 | 🞏 | 🞏 | 🞏 | 🞏 | 🞏 | 🞏 | 🞏 |
| 1. Health and Safety Manager | 🞏 | 🞏 | 🞏 | 🞏 | 🞏 | 🞏 | 🞏 | 🞏 | 🞏 | 🞏 | 🞏 |
| 1. Health and Safety Representative | 🞏 | 🞏 | 🞏 | 🞏 | 🞏 | 🞏 | 🞏 | 🞏 | 🞏 | 🞏 | 🞏 |
| 1. Workers | 🞏 | 🞏 | 🞏 | 🞏 | 🞏 | 🞏 | 🞏 | 🞏 | 🞏 | 🞏 | 🞏 |
| 1. Occupational Physician | 🞏 | 🞏 | 🞏 | 🞏 | 🞏 | 🞏 | 🞏 | 🞏 | 🞏 | 🞏 | 🞏 |

# How useful do you think are the following types of medical visits foreseen by Legislative Decree 81/08 and subsequent amendments and additions, for the maintenance of the job of a worker with cancer? (scale from 0 = useless to 10 = completely useful)

|  | **0**  **Useless** | **1** | **2** | **3** | **4** | **5** | **6** | **7** | **8** | **9** | **10**  **Completely useful** |
| --- | --- | --- | --- | --- | --- | --- | --- | --- | --- | --- | --- |
| 1. Preventive | 🞏 | 🞏 | 🞏 | 🞏 | 🞏 | 🞏 | 🞏 | 🞏 | 🞏 | 🞏 | 🞏 |
| 1. Estimate in the pre-employment phase | 🞏 | 🞏 | 🞏 | 🞏 | 🞏 | 🞏 | 🞏 | 🞏 | 🞏 | 🞏 | 🞏 |
| 1. Change of task | 🞏 | 🞏 | 🞏 | 🞏 | 🞏 | 🞏 | 🞏 | 🞏 | 🞏 | 🞏 | 🞏 |
| 1. Periodic | 🞏 | 🞏 | 🞏 | 🞏 | 🞏 | 🞏 | 🞏 | 🞏 | 🞏 | 🞏 | 🞏 |
| 1. Requested by the worker | 🞏 | 🞏 | 🞏 | 🞏 | 🞏 | 🞏 | 🞏 | 🞏 | 🞏 | 🞏 | 🞏 |
| 1. Prior to return to work after 60 continuous days of absence for health reasons | 🞏 | 🞏 | 🞏 | 🞏 | 🞏 | 🞏 | 🞏 | 🞏 | 🞏 | 🞏 | 🞏 |

**SECTION D. Occupational Physicians training and updating needs on issues regarding the management of workers affected by cancer**

# How much do you agree with the statement: a specific training in the field of cancer and work is important for the Occupational Physician?

# Completely disagree

# Mostly disagree

# Slightly agree

# Mostly agree

# Completely agree

# How useful do you think are the following issues for your training and update needs on cancer and work? (scale from 0 = useless to 10 = completely useful)

|  | **0**  **Useless** | **1** | **2** | **3** | **4** | **5** | **6** | **7** | **8** | **9** | **10**  **Completely useful** |
| --- | --- | --- | --- | --- | --- | --- | --- | --- | --- | --- | --- |
| 1. Health surveillance protocols | 🞏 | 🞏 | 🞏 | 🞏 | 🞏 | 🞏 | 🞏 | 🞏 | 🞏 | 🞏 | 🞏 |
| 1. Criteria for formulating the fitness judgment | 🞏 | 🞏 | 🞏 | 🞏 | 🞏 | 🞏 | 🞏 | 🞏 | 🞏 | 🞏 | 🞏 |
| 1. Occupational risks | 🞏 | 🞏 | 🞏 | 🞏 | 🞏 | 🞏 | 🞏 | 🞏 | 🞏 | 🞏 | 🞏 |
| 1. Risk assessment | 🞏 | 🞏 | 🞏 | 🞏 | 🞏 | 🞏 | 🞏 | 🞏 | 🞏 | 🞏 | 🞏 |
| 1. Emergency management | 🞏 | 🞏 | 🞏 | 🞏 | 🞏 | 🞏 | 🞏 | 🞏 | 🞏 | 🞏 | 🞏 |
| 1. Reasonable accommodations | 🞏 | 🞏 | 🞏 | 🞏 | 🞏 | 🞏 | 🞏 | 🞏 | 🞏 | 🞏 | 🞏 |
| 1. Cancer Clinic | 🞏 | 🞏 | 🞏 | 🞏 | 🞏 | 🞏 | 🞏 | 🞏 | 🞏 | 🞏 | 🞏 |
| 1. Cancer Therapy | 🞏 | 🞏 | 🞏 | 🞏 | 🞏 | 🞏 | 🞏 | 🞏 | 🞏 | 🞏 | 🞏 |
| 1. Rehabilitation courses | 🞏 | 🞏 | 🞏 | 🞏 | 🞏 | 🞏 | 🞏 | 🞏 | 🞏 | 🞏 | 🞏 |
| 1. Welfare legislation | 🞏 | 🞏 | 🞏 | 🞏 | 🞏 | 🞏 | 🞏 | 🞏 | 🞏 | 🞏 | 🞏 |
| 1. Social security legislation | 🞏 | 🞏 | 🞏 | 🞏 | 🞏 | 🞏 | 🞏 | 🞏 | 🞏 | 🞏 | 🞏 |
| 1. Legislation on targeted placement | 🞏 | 🞏 | 🞏 | 🞏 | 🞏 | 🞏 | 🞏 | 🞏 | 🞏 | 🞏 | 🞏 |
| 1. Medical-legal requirements | 🞏 | 🞏 | 🞏 | 🞏 | 🞏 | 🞏 | 🞏 | 🞏 | 🞏 | 🞏 | 🞏 |
| 1. Counseling to the worker on the rights due | 🞏 | 🞏 | 🞏 | 🞏 | 🞏 | 🞏 | 🞏 | 🞏 | 🞏 | 🞏 | 🞏 |

# How useful do you think are the following teaching methodologies, for your training? (scale from 0 = useless to 5 = completely useful).

|  | **0**  **Useless** | **1** | **2** | **3** | **4** | **5**  **Completely useful** |
| --- | --- | --- | --- | --- | --- | --- |
| 1. Training courses with frontal lessons | 🞏 | 🞏 | 🞏 | 🞏 | 🞏 | 🞏 |
| 1. Practical exercises | 🞏 | 🞏 | 🞏 | 🞏 | 🞏 | 🞏 |
| 1. Analysis and cases discussion | 🞏 | 🞏 | 🞏 | 🞏 | 🞏 | 🞏 |
| 1. Congresses/Conferences | 🞏 | 🞏 | 🞏 | 🞏 | 🞏 | 🞏 |
| 1. Role-playing | 🞏 | 🞏 | 🞏 | 🞏 | 🞏 | 🞏 |
| 1. Remote training | 🞏 | 🞏 | 🞏 | 🞏 | 🞏 | 🞏 |
| 1. Self-learning courses (e-learning) | 🞏 | 🞏 | 🞏 | 🞏 | 🞏 | 🞏 |
| 1. Self-learning courses (paper support) | 🞏 | 🞏 | 🞏 | 🞏 | 🞏 | 🞏 |
| 1. Workshops | 🞏 | 🞏 | 🞏 | 🞏 | 🞏 | 🞏 |
