# Supplementary figures and images for: The challenges of managing patients with cancer in the workplace: Needs, opportunities and perspectives of occupational physicians
Source: PLoS One. 2023 Jul 27;18(7):e0288739. doi: 10.1371/journal.pone.0288739 (PMC10374089; doi:10.1371/journal.pone.0288739)

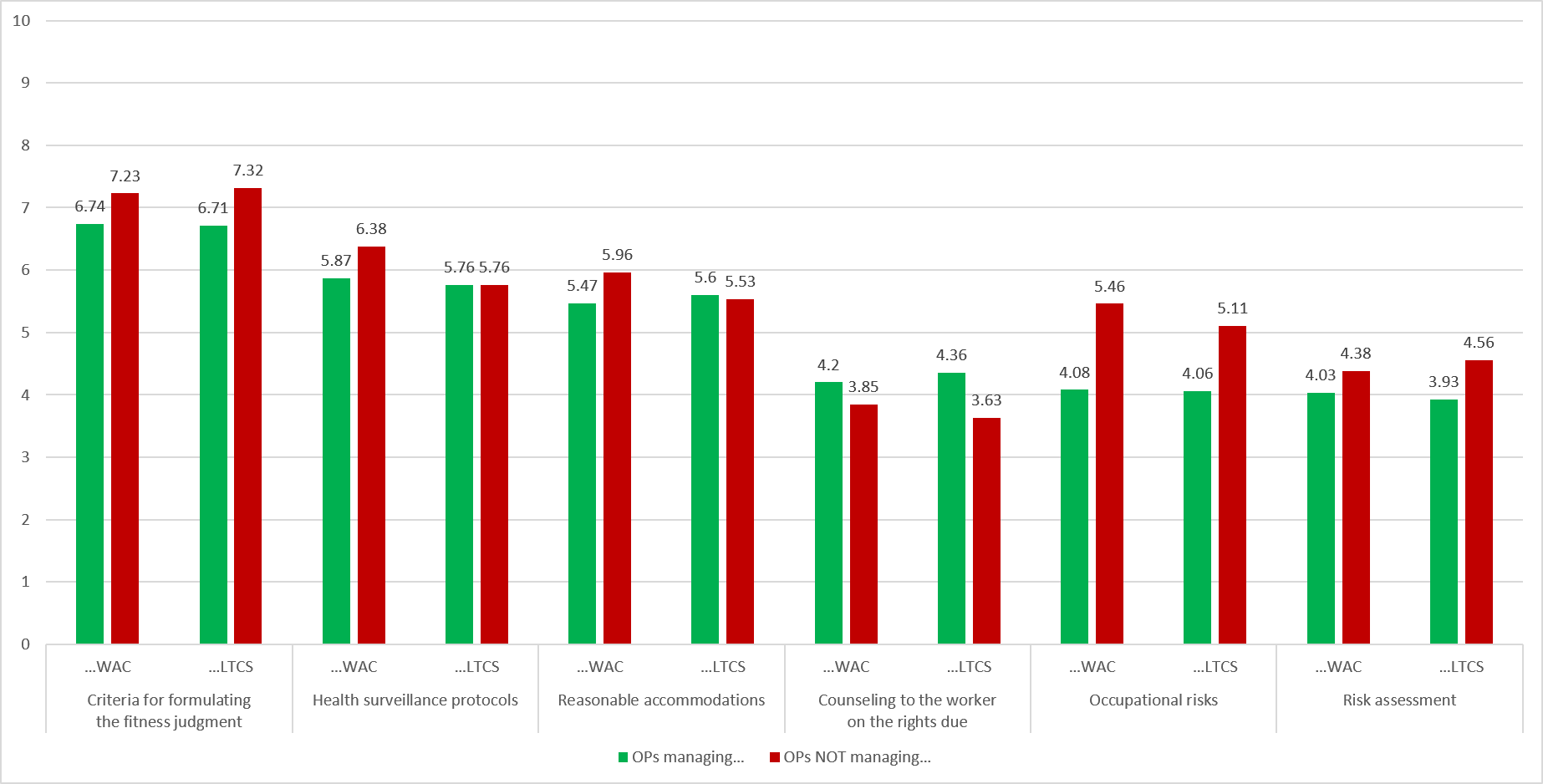

Supplement: S1 Fig — (TIF) [file pone.0288739.s003.tif]

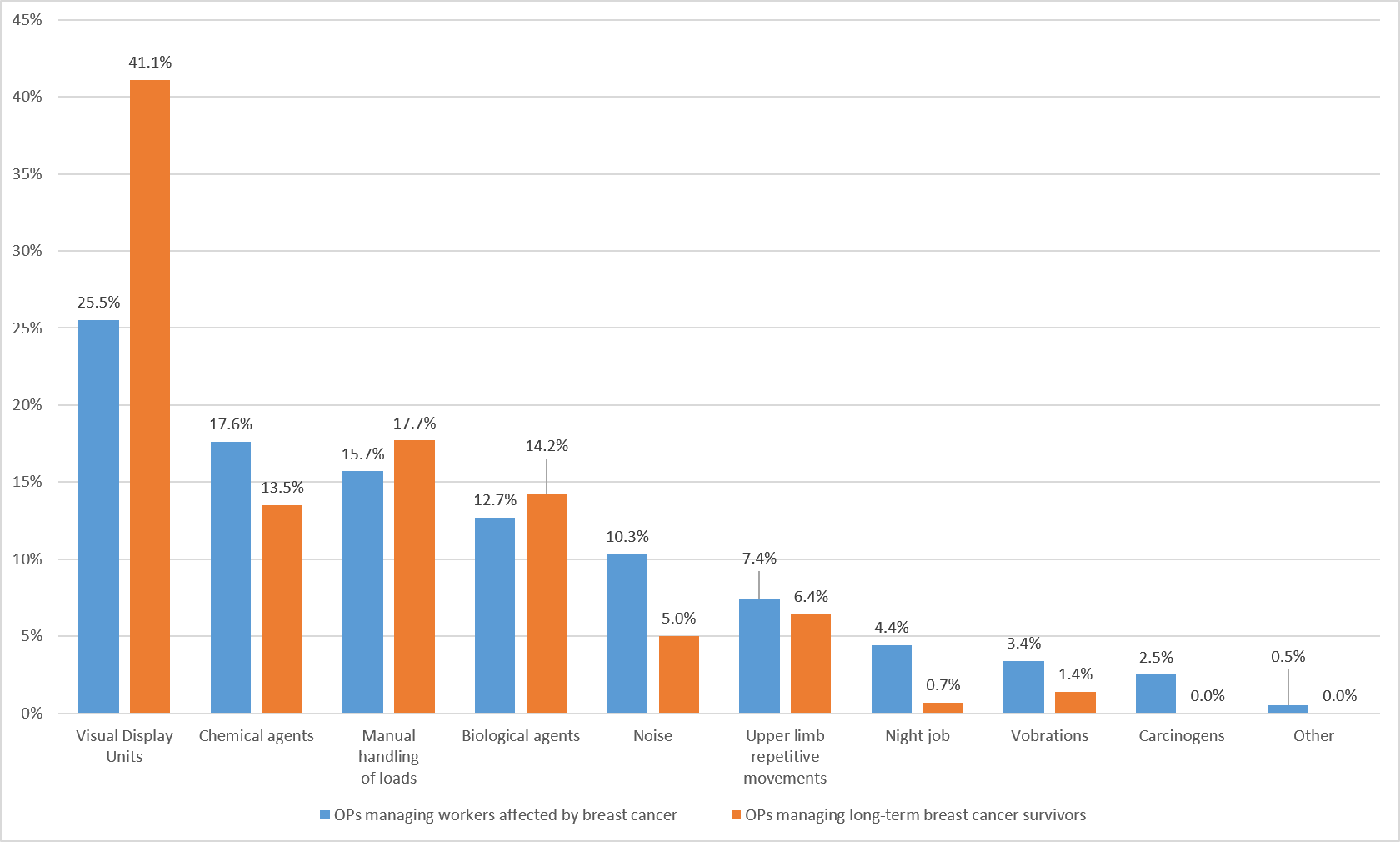

Supplement: S2 Fig — Multiple choice question. Percentages of answers. (TIF) [file pone.0288739.s004.tif]
